# Relative Deprivation *and* Relative Wealth Enhances Anti-Immigrant Sentiments: The V-Curve Re-Examined

**DOI:** 10.1371/journal.pone.0139156

**Published:** 2015-10-13

**Authors:** Jolanda Jetten, Frank Mols, Tom Postmes

**Affiliations:** 1 University of Queensland, Brisbane, Australia; 2 University of Groningen, Groningen, The Netherlands; University of Westminster, UNITED KINGDOM

## Abstract

Previous research has shown that negative attitudes towards immigrants and support for anti-immigrant parties are observed both among those experiencing relative deprivation *and* those experiencing relative gratification (so called v-curve). Whereas the effect of relative deprivation is intuitive, the effect of relative gratification is more difficult to explain. Why would economic prosperity provoke negative attitudes towards immigrants? We first present correlational (Study 1) and experimental (Study 2) support for the v-curve. In Study 1, in a national Swiss referendum, a higher percentage anti-immigrant voting was found in cantons with relatively lower and relatively higher relative disposable income. In Study 2, in a hypothetical society, more opposition to ‘newcomers’ joining society was found among poor or above average wealth group members than among those in a moderate wealth group condition. In Study 3, we replicate this finding and also show that opposition to immigration is higher for all wealth groups when societal inequality is growing rather than declining. In a final study, we examine different forms of relative gratification and mediators of the relationship between relative gratification and opposition to immigration (i.e., identification, collective self-definition as competent and cold, and fear about future wealth). Only fear about future wealth mediates this relationship. We conclude that, paradoxically, relative gratification effects are partly due to the fear of future deprivation.

## Introduction

Groups openly advocating anti-immigrant sentiments have made a remarkable comeback in recent years in OECD countries. This trend, which began in the 1980s, can even be witnessed in countries where multiculturalism was once celebrated as a core value defining the national identity. Consider the Netherlands, where the PVV (Party for Freedom) led by Geert Wilders increased its number of seats in parliament from 9 seats in 2006 to 24 in the 2010 elections. Another case in point is Sweden, where, for the first time in the country’s history, the extreme right secured a seat in the national parliament in the 2010 elections.

Attempts to understand the rise of support for anti-immigrant parties traditionally focus on macro-economic downturn (such as the 1930s Great Depression and rapid post-war industrial modernization) and poor people or working class people. This is because realistic conflict and competition over scarce resources is experienced most strongly in such an economic climate [1], and among those with a lower economic position in society [2–7]. While we do not dispute that economic downturn or poverty can contribute to anti-immigrant sentiments, there is a growing body of work that suggests that this relationship is not as straightforward as often believed. In particular, while there is considerable evidence that economic downturn *can* be associated with harsh attitudes towards immigrants, there is also evidence for the opposite: that economic prosperity can produce harsh attitudes towards minorities—so called *relative gratification* [8]. Similarly, while negative attitudes towards minorities are at times more pronounced among the poor and working classes, such attitudes are under some conditions particularly prevalent among the wealthier groups in society, or those who expect prosperity in the future [9–11]. Indeed, while support for anti-immigrant parties flourished after the Global Financial Crisis (GFC), many commentators have noted that the rise of support for extreme right in Western Europe started long before the GFC—at a time when the economy in most Eurozone countries was booming and, relatively speaking, people saw their income and affluence increase [12–14].

To illuminate these opposing processes, we take a two-pronged approach. We start by demonstrating empirically that *both* relative deprivation and relative gratification processes relate to opposition to immigration. Second, in an attempt to better understand relative gratification processes, we examine (a) whether the v-curve is affected by socio-structural changes relating to societal inequality, (b) the forms of relative gratification that are related to opposition to immigration, and (c) mediators underlying the relationship between relative gratification and opposition to immigration. In line with Guimond and Dambrun [9], we define relative deprivation as a group’s perception that it is deprived in comparison to a particular standard or compared to a relevant outgroup. In contrast, relative gratification is the experience that the group is better off compared to a particular standard or a relevant other group. We begin by reviewing past research on the way economic performance affects prejudice and opposition to immigration.

### Hard times = harsh attitudes?

The idea that economic hardship fuels negative attitudes towards minorities forms the basis for theorizing in social psychology, political science and sociology. Both realistic conflict ([15–17]; see [18] for a meta-analysis) and relative deprivation theorising [19] leads to the predication that, when confronted with economic hardship, people ‘lash out’ at minorities—in particular those minorities who appear to be competing for resources or are perceived to be the cause of relative deprivation (see also frustration-aggression reasoning and scapegoat theory, [20]). Consistent with this, political economy researchers have argued that attitudes towards immigrants are conditioned by the extent to which immigrants are perceived to threaten material self-interest [21, 22].

At a societal level, there is considerable evidence for this relationship, both in Europe [23] and in North America [24, 25]. Economic downturns have also been associated with an increased propensity to vote for parties voicing anti-immigration sentiments and an increase in anti-immigrant attitudes more generally [4, 26–34]. There is now also a considerable body of literature showing that poor uneducated manual workers are one category of voters to be drawn to extreme right wing parties with anti-immigrant agendas [27, 31, 35] and to hold more negative attitudes towards immigrants than their wealthier counterparts [6]. In sum, it appears that it is during harsh economic times, and among income groups experiencing harsh conditions that we encounter harsh attitudes towards minorities.

However, even though such findings appear to explain *some* instances of prejudice, it is also clear that there are many cases where realistic conflict, self-interest and relative deprivation theorising fail to explain opposition to immigration. For one, some claims are poorly supported on closer inspection [36–38]. Indeed, on the basis of their review of the literature in the field, Hainmueller and Hopkins [37] conclude that “the significant majority of prior work finds that labor market competition does not shape attitudes [towards immigrants] of the mass public” (p.240). This state of affairs led to Ceobanu and Escandell [39] to recommend that it is time to move beyond theorising that explains anti-immigrant attitudes solely by pointing to individual and group-level competition and threat.

It is also worth considering classic work by Hovland and Sears [40] purportedly showing that in the period between 1882 and 1930, low cotton prices were associated with a higher number of lynchings of Blacks in the Southern states in the US. A re-analysis of this phenomenon, taking account of a larger time period, failed to replicate this relationship between economic performance and hostility towards minorities [41]. There is also work showing no relationship between economic downturn or wealth of groups in society and support for political parties with an anti-immigrant agenda. For example, Lubbers and Scheepers’ [31] analysis of opinion poll data collected in different German regions between 1989 and 1998 shows that there is no straightforward correlation between a region’s economic performance and support for the anti-immigrant party, the Republikaner. Consistent with this, Hannah Arendt, in her classic book ‘The Origins of Totalitarianism’ [42] points out that totalitarian movements developed in Germany and Italy, but not in the United Kingdom, despite the fact that both countries suffered to the same extent from the consequences of the Great Depression ([42], p. 250–254; see also [43]).

More interesting for our present purposes, research examining the electoral support for Hitler’s National Socialists (NSDAP) in different regions of the Weimar Republic found that support for the party was indeed strongest in depressed working-class regions (e.g. Thuringia) but also in relatively affluent protestant rural areas such as Schleswig-Holstein, Mecklenburg, Pomerania and East Prussia [44]. Likewise, a study examining exceptionally high levels of support for the NSDAP in the city of Braunschweig in the early 1930s (between 36 and 36.6% of the vote), revealed that support for the NSDAP was highest in the seven upper middle-class precincts of the city, with levels of support ranging from 61% to 65.5% [45]. Similarly, Vanneman and Pettigrew [8] found that in Chicago, resistance to black mayor candidates was highest among those who were fraternally deprived and among more affluent workers.

The notion that sometimes it is groups at the top of the hierarchy (i.e., high status groups) that show most negative intergroup attitudes is also consistent with findings of a meta-analysis by Bettencourt, Dorr, Charlton, and Hume [46]. Their research, conducted in both real-world and laboratory settings, showed that high-status groups generally display more ingroup bias than do low-status groups. It has also been found that higher status groups are more likely than lower status groups to endorse group-based hierarchies and status-inequalities [47, 48], and that those with greater wealth donate less generously, score lower in empathic accuracy, and are less responsive to the needs of others than their poorer counterparts [49–52]. Similar effects have been witnessed at the societal level (see [53], in press). In a study in which the state of the national economy was experimentally manipulated, stronger anti-immigrant sentiments were found when the national economy was presented as prospering rather than contracting. These findings are also consistent with research testing Labor Market Competition Theory predictions [54]. Researchers working in this research tradition have suggested that opposition to immigration can be expected to be higher among the more skilled and the wealthier segments in society. This is because these groups are most concerned about increased immigration being a burden on the public purse, and factor increasing pressure to raise taxes.

Other work suggests that these effects are most pronounced when high status group members perceive their high status to be unstable or insecure. High status group members whose status is threatened in this way might engage in status protection, or even oppression to avoid losing their higher status. For example, Haslam [55] argues that under such conditions, high-status group behavior is “likely to take a more sinister form and be reflected in ideologies (racism and sexism for example) that attempt to justify and rationalize the ingroup’s superiority and the outgroup’s inferiority” (p.27). Consistent with this, Scheepers, Ellemers, and Sintemaartensdijk [56] showed experimentally that only high status groups who feared to lose their status in the future displayed a stress response in the form of higher systolic blood pressure and pulse pressure.

Similarly, Rios Morrison, Fast and Ybarra [57] found that members of high status groups who were more highly identified with their group were only more likely to endorse inequality between groups when they experienced high intergroup threat. More generally, sociologists have argued that high-status groups perceive threats to their status position when they feel that they might lose the concrete privileges to lower-status groups (e.g., [58, 59]). Indeed, it has been argued that it is the fear that one compares negatively to other relevant groups, concerns that others are climbing the ladder faster than oneself, or anxiety related to the belief that one’s earlier economic gains might be lost that feeds discontent and hostility towards minorities ([60], see also [61]).

According to Blumer [58], prejudice arises when a privileged group develops fear that a minority group is ‘getting out of place’ and ‘encroaches’ on benefits that are typically seen as benefits of their group [59, 62]. In line with this notion, a study by Martinovic and Verkuyten [63] found that endorsement of the belief that primo occupants are more entitled than those arriving later (i.e., autochthony) was related to prejudice towards immigrants only for Dutch natives who feared that they were losing their entitlements to newcomers.

In sum, realistic conflict theorizing may be popular and intuitively appealing, the empirical evidence for the theory is rather mixed and inconclusive: negative attitudes towards minorities have both been observed in times of economic hardship *and* in times of economic prosperity; among members of low status groups as well as among those who belong to high status groups. While realistic conflict literature accounts well for the finding that economic hardship enhances harsh attitudes, theoretically, it cannot account for the finding that at times, we appear to be particularly harsh towards minorities such as immigrants when our own economic, financial and social status is relatively high, or when a society’s economy is booming.

### How economic prosperity hardens attitudes

Researchers examining the so-called v-curve developed the notion that both relative deprivation *and* relative gratification may underlie prejudice and negative attitudes towards minorities. Specifically, negative attitudes towards minorities such as immigrants are predicted to be highest among those who experience relative deprivation (because of low status or economic hardship in society) and among those who experience relative gratification (either because their status is high or because they experience economic prosperity more generally, [9], see also [64]. There is now a large body of work that has provided empirical evidence in line with the v-curve, both in real world contexts [65] as well as in more controlled experimental studies [9, 66, 11].

Previous research has offered a number of explanations for the finding that relative gratification is associated with opposition to immigration and more intergroup discrimination. Dambrun and colleagues [65] reasoned that relative gratification enhances the attractiveness of the ingroup, and this, so they argued, is associated with stronger intergroup dynamics, which translate into more intergroup discrimination. Consistent with this, they found that group identification partially mediated the relationship between relative gratification and negative intergroup attitudes in South-Africa. Likewise, Postmes and Smith [11] found evidence for the mediating role of group identification. Importantly however, in these studies it cannot be argued that it is identification *with the income group* that is mediating the effect. Dambrun and colleagues [65] measured identification in relation to participants’ ethnicity while relative gratification was assessed in relation to personal economic conditions. Postmes and Smith [11] manipulated relative gratification as favourable job prospects for university students while identification referred to identification with Britain.

In a further exploration of processes underlying the v-curve, Guimond and Dambrun ([9], Study 2) manipulated relative gratification by providing feedback that job prospects of the ingroup were more favourable in the future than that of another comparison group. They found the predicted v-cure but failed to find evidence that social dominance or mood mediated the effect of relative gratification on prejudice and support for anti-immigrant policies. More recently, Moscatelli and colleagues [66] identified two mediators underlying the relationship between relative gratification and intergroup discrimination. First, relative gratification was found to lead to a fear of losing ingroup advantage, and this in turn was associated with greater intergroup discrimination. Second, relatively gratified groups were found to feel guilty about the advantage they had over relatively deprived groups. The authors reasoned that such feelings justified a “strike first” attitude, whereby intergroup discrimination is shown because the outgroup in question is expected to be biased against the relatively gratified ingroup responsible for their deprived state.

While these lines of work provide some important insights into the mechanisms underlying the effect of relative gratification on opposition to immigration, a few considerations lead us to suspect that the mediation question has not yet been resolved. First, not all studies that examine the underlying processes are concerned with opposition towards immigration (or outcomes that are related to this dependent variable). For instance, Moscatelli et al. [66] manipulated relative gratification of two groups in relation to one another. In their study, participants allocated resources to their own privileged group and to the disadvantaged group. Even though this setting enabled the researchers to gain a deeper understanding of competition between two rival groups, it is not a proper test of the effects of gratification on hostility towards immigrants. After all, immigrants (and in particular potential future immigrants) are not necessarily in direct competition with privileged members of society. Thus, this work cannot help us to understand how relative gratification shapes attitudes towards a third party (e.g., immigrants). In order to make progress in this line of research, it is important to note an important difference. Whereas Moscatelli and colleagues found that guilt was an important mediator of the relationship between relative gratification and intergroup discrimination, these effects are probably restricted to contexts where relative gratification was achieved at the expense of the outgroup [66]. In our research, we therefore measure and manipulate relative gratification independently from the group to be evaluated (e.g., newcomers or immigrants).

Second, rather than examining consequences of *present* gratification, Guimond and Dambrun [9] manipulated relative gratification as the likelihood of *future* gratification (e.g., job prospects for undergraduate students after finishing their degree) and are thus concerned with the behaviour of *prospective* group members [11]. In our studies, we examine the effects of relative gratification as a current state, rather than as one that can be expected in the future. We also build on this work by examining whether different forms of relative gratification (i.e., present vs. future; personal versus group) relate differently to opposition to immigration.

In an attempt to gain a better understanding of the processes underlying relative gratification effects, we examine the mediating role of a number of different variables. First, similar to Dambrun et al. [65] we explore the extent to which identification with the wealth group mediates the effects of relative gratification on opposition to immigration. Second, we examine whether relative gratification effects are mediated by collective self-definitions. Specifically, and in line with stereotype content model [67, 68] we propose that the stereotype of the wealthy—because of this group’s ability to acquire wealth—tends to revolve around attributes associated with competence. However, wealthy groups are also seen as relatively cold (i.e. low in empathy). These stereotypes are not just ways in which others perceive the affluent, they are also internalized by the wealthy themselves as ways to understand the group’s identity, affecting the content of group norms [11]. We expect that the relatively prosperous groups will self-stereotype as competent but cold, the more group norms will emerge that condone and justify hostility and opposition towards those aspiring to become part of society (i.e., immigrants).

Finally, we examine the extent to which relative gratification effects are due to so-called ‘fear of falling’ [69]. In line with Moscatelli and colleagues [66] we predicted that with increasing levels of relative wealth, anxiety related to the belief that one’s earlier economic gains might be lost may feed discontent and hostility towards minorities [61]. This reasoning is consistent with social identity theorizing that anxiety about the security of high status will enhance status protection behaviour and enhance the motivation to justify the current status quo [55, 56, 70].

### Overview of Research

We conducted four studies to examine support for our hypotheses. First, in all studies, we examined support for the v-cure hypothesis. We predicted that compared to moderate levels of wealth, lower and higher wealth will be associated with more opposition to immigrants and towards immigration policies. In a first study, involving a Swiss referendum, we examined whether percentages of votes in favour of curbing immigration would be higher in cantons that are relatively poor *and* cantons that are relatively prosperous (compared to intermediate levels of wealth). In Study 2, in an attempt to provide evidence for causality, we tested the same v-curve prediction using an experimental paradigm where we systematically manipulated the position of the group in the hierarchy (poor, moderately wealthy and wealthy).

In a third study, we examined whether features of the socio-structural context affect the prevalence of the v-curve. Specifically, we examined whether the extent to which relative gratification enhances opposition to immigration is affected by growing inequality in society. Inequality has been associated with the perception that society is hostile, and, building on previous work that has shown that relative gratification is only associated with more hostility towards immigrants when group norms condone such harshness [11], we predicted that the v-curve will be more pronounced in conditions where societies face growing inequality between wealth groups (compared to declining inequality).

In a final study, we examined the v-curve in a representative sample of Australians. This study had several additional aims. First, we examined different forms of relative gratification and examine for instance whether effects of past, current and future gratification on opposition to immigration differ and whether perceived personal relative gratification effects are similar to those of collective relative gratification. No strong predictions were formulated, but we were open to the finding that because relative gratification is felt more strongly when it is a current than an anticipated state, the former relative gratification form would be stronger predictor of opposition to immigration than the latter. Furthermore, in line with classic reasoning that collective level responses are best predicted by fraternalistic deprivation [71, 8], we expected that collective relative gratification (i.e., fraternalistic) would be a better predictor of opposition to immigration than personal gratification (i.e., egoistic).

In addition, in this study we systematically examined support for the proposed mediators of the relationship between collective relative gratification and opposition to immigration. We focused on (1) wealth group identification, (2) collective self-definition as competent but cold, and (3) fear for the future wealth of Australia as mediators to the relationship between relative gratification and opposition to immigration.

## Study 1

### Ethics Statement

Because no data were collected among human participants, and because data were obtained from publicly available data bases, it was not necessary to obtain ethical clearance for this study.

### Method

On February 9, 2014, a referendum was held in Switzerland asking the Swiss to vote on the question whether they agreed (“yes”) or disagreed (“no”) that immigration into the country should be curbed. A small majority of Swiss citizens (50.3%) agreed with the proposal requiring the government to set an upper immigration limit. However, despite the acceptance of the proposal nationally, levels of endorsement within the 26 cantons varied considerably with ‘only’ 38.90% “yes” votes in Vaud and 68.20% “yes” votes in Ticino (see [72, 73] for more information on this referendum).

A data set was created bringing together information on (a) anti-immigrant voting by canton (we included the percentage “yes” votes, and the total number of “yes” votes) and (b) indicators of the canton’s economic performance (we included 2 indicators: percentage unemployment in the canton in 2013 and the canton’s relative disposable income in 2011).

### Results

Descriptive statistics, bivariate correlations and partial correlations controlling for total number of votes in a canton and seats in the parliament can be found in Table 1. Inspection of the partial correlations shows relative gratification relationships: a higher percentage of “yes” votes was negatively correlated with unemployment, *r* (26) = -.50, *p* = .012, and positively correlated with relative disposable income, *r* (26) = .57, *p* = .004. The relationship between percentage of “yes” votes and the quadratic term for unemployment was also significant, *r* (26) = -.47, *p* = .019, but non-significant for relative disposable income, *r* (26) = −049, *p* = .820. Inspection of the curvilinear effect for unemployment showed that the percentage of “yes” votes was higher in cantons with relatively low and relatively high levels of unemployment (compared to the cantons with relatively moderate levels of unemployment).

**Table 1:** Descriptive statistics, bivariate correlations and partial correlations, votes to curb immigration by canton in Switzerland, Study 1.

	*M (SD)*	1	2	3	4	5	6
1. “Yes” votes (%)	52.55 (8.00)	1	-.50*	-.47*	.57**	-.05	
2. Unemployment (%)	2.54 (1.18)	-.62**	1	—	-.63**	.18	
3. Squared Unemployment (%)	7.81 (6.99)	-.60**	—	1	-.67**	.25	
4. Relative disposable income	.53 (1.26)	.71**	-.72**	-.75**	1	—	
5. Squared Relative disposable income	1.82 (3.03)	-.22	.28	.36	—	1	
6. Total votes by canton	111860.85 (118841.66)	.27	.28	.22	-.36	-.12	1
7. Seats in national parliament	7.69 (8.07)	-.31	.31	.25	-.41*	-.07	.99**

*p < .05

**p < .01

Bivariate correlations are presented below the diagonal. The partial correlation controlling for total number of ‘yes’ votes is reported above the diagonal. Correlations are based on *N* = 26.

### Discussion

In sum, it was in cantons with relatively lower levels of unemployment and relatively higher disposable income that the percentage of “yes” votes was higher. For the relationship between percentage of “yes” votes and unemployment, we also found evidence of a v-curve whereby this negative relationship was relatively speaking more pronounced in the poorer and the wealthier cantons compared to the moderately wealthy cantons.

However, when exploring canton effects, the sample size is by definition small and this affects the certainty with which we can draw conclusions. Furthermore, although this case study provides preliminary evidence for a v-curve, this evidence needs to be interpreted with caution because a host of other variables could potentially have affected “yes” votes other than those relating to unemployment and relative disposable income. Therefore, in Study 2 we used an experimental design allowing us to assess whether relative deprivation and relative gratification *causes* differences in opposition to immigration.

## Study 2

We developed an experimental paradigm that would allow us to isolate effects and examine causality. We again tested support for a v-curve between wealth and opposition to immigration in an experiment in which participants joined a stratified virtual society, and were subsequently asked for their attitudes towards ‘newcomers’ (i.e., immigrants) who would be joining their society.

### Participants and Design

Participants were 61 undergraduate students at a large Australian university (48 females, 13 males with an average age of *M =* 18.82 years, *SD* = 2.81). Australian citizenship was a prerequisite for participation in the study. Participants were randomly assigned to one of three wealth conditions: as poor (i.e., lower than average wealth), moderate wealth, or above average wealth.

### Ethics Statement

This study obtained ethical clearance from the Behavioural and Social Sciences Ethical Review Committee (BSSERC) at the University of Queensland. Before completing the questionnaire, participants were informed about the aims of the study. After this, they were informed that continuing with the survey indicated (written) informed consent.

### Procedure and measures

Participants were informed that they would become part of a virtual society, Bimboola, and that they were starting a new life as a member of this society (for more information on the paradigm, please contact the first author). Participants read that, just like any other society, there are differences in income within this society. They were informed that Bimboola consisted of 5 income groups, with group 1 earning less than 5,000 Bimbolian Dollars (BD) per year (below the poverty threshold), group 2 earning between 5,000 and 10,000 BD, group 3 earning between 10,000 and 100, 000 BD per year, group 4 earning between 100,000 and 1 million BD per year and group 5 earning more than 1 million BD per year. Participants were allocated to income group 2, 3, or 4 and they were told that allocation was random. Income groups 1 and 5 were primarily included to provide a broader frame and to avoid that income group 2 and 4 would also be the poorest or the wealthiest within the society. In this study, participants were allocated to income group 1and 5 but these results will not be further discussed because in these conditions income level is confounded with an extreme income position (i.e., being at the bottom or top of the wealth hierarchy).

Once participants knew which income group they belonged to, they were invited to start their new life. Their first task was to purchase basic requirements such as a house, a car, and a phone. When choosing a house, participants were shown pictures of houses ranging from old and run-down dwellings to brand-new luxurious mansions. The houses on offer were displayed on screen, and listed by income group (ranging from 1 to 5). Participants were advised that they could only buy a house within or below their income bracket. The same procedure was used when buying a car and phone. Participants were then told they had the opportunity to select a holiday destination. Whereas the holiday options for income group 2 were quite basic (e.g., camping or a day-trip to the beach), income group 3 could go on a 4-wheel drive adventure, a week-long campervan trip, or a one week stay in a lakeside cottage. Participants in income group 4 could choose from any of three more extravagant options such as a one-week shopping trip to Paris.

Participants completed a manipulation check asking them to indicate to what extent they agreed with the statements “my group is poor” and “my group is rich” on a 7 point scale ranging from 1 = *Strongly disagree* to 7 = *Strongly agree*. Participants were then informed that a new group (called newcomers) was about to join Bimboola. They were told that these newcomers wanted to rebuild their lives in Bimboola and that they may need some assistance from existing members of the community of Bimboola. After this, opposition to immigration by these newcomers was measured. After completing all measures, participants were debriefed and thanked for their participation.

#### Opposition to immigration

To measure opposition to immigration by the newcomers, 21 items measuring both symbolic (e.g., “The cultural practises of the new group will threaten the Bimboolean way of life”) and realistic threat perceptions (e.g., “The presence of people from this new group will increase unemployment in Bimboola”) and well as general items (e.g., “I think our group should not allow newcomers to Bimboola”) were included. Responses were measured on a 7 point scale from 1 = *Strongly disagree* to 7 = *Strongly agree* (α = .91).

### Results

#### Manipulation check

The two manipulation check items were combined after reverse scoring the statement “I am poor” so that higher scores on this measure indicate the perception that the own income group is wealthy (*r* = .77, *p* < .001). One-way analysis of variance on the wealth manipulation check revealed that the experimental conditions differed significantly from one another, *F*(2, 58) = 70.60, *p*<. 001. In line with the manipulations, above average wealth income group participants agreed most with the statement that their group was rich (*M* = 6.29, *SD* = .75), followed by moderately wealthy income group participants (*M* = 4.39, *SD* = 1.01), followed by poor income group participants (*M* = 2.34, *SD* = 1.13). Post-hoc tests confirmed that all conditions differed significantly from each other at *p* < .001.

#### Opposition to immigration

**Fig 1:**
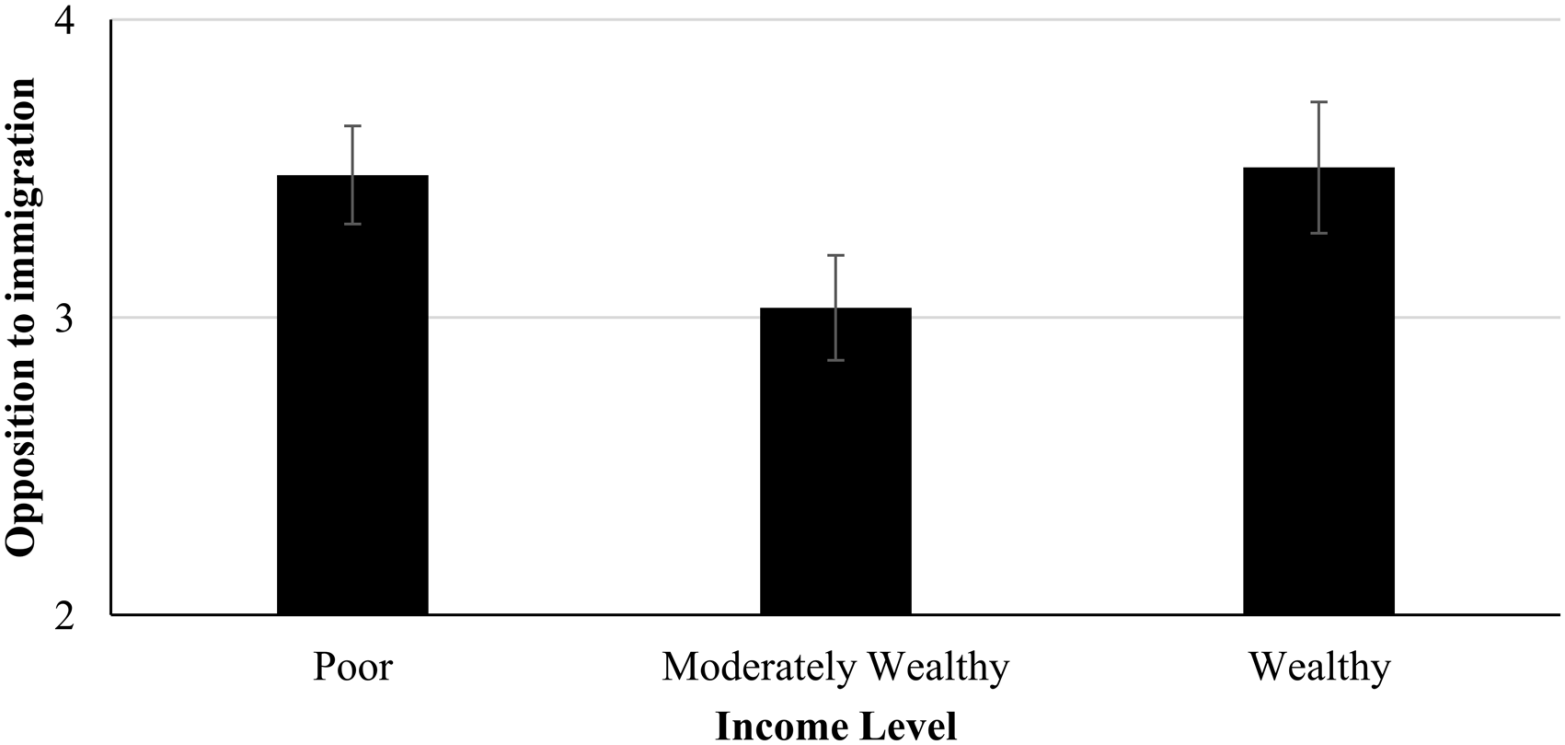
Opposition to immigration as a function of income level, Means and Standard Errors, Study 2.

A one-way analysis of variance was set up testing the linear as well as the curvilinear condition effect on opposition to immigration. The linear effect was not significant, *F*(1, 58) = .07, *p* = .79, but the quadratic term was significant, *F*(1, 58) = 4.18, *p* = .045. Opposition to immigration was highest for the poor income group (income group 2, *M* = 3.48, *SD* = .80) and the above average wealth income group (income group 4, *M* = 3.50, *SD* = .73) and lowest for the moderately wealth group (income group 3, *M* = 3.03, *SD* = .91, see Fig 1).

### Discussion

In sum, results of Study 2 showed that the developed experimental design is effective in manipulating wealth perceptions and that the own group’s wealth perceptions *cause* opposition to immigration differences. Specifically, we found a v-curve relationship whereby it is those in poor *and* above average wealth income groups who were most opposed to immigration compared to those in a moderately wealth group.

## Study 3

Taking Study 2 findings further, in Study 3 we examined the way the broader structural context affects the v-curve, using a similar ‘new life’ experiment as used in Study 2. In particular, and building on research by Postmes and Smith [11] who showed that the v-curve was only found when the broader social context endorsed a hostile normative climate, we predicted that inequality would amplify the v-curve. In line with findings that societal inequality is associated with low trust, low cohesion, a harsher and more hostile society [74] and a perceived loss of control [75]—all factors that impact on the perceived stability of wealth—we predict that in particular those assigned to poor and above average wealth groups would be affected by the instability that inequality brings (more so than those assigned to moderately wealth conditions). Importantly, we manipulated *perceived inequality* in such a way that the level of wealth of the participant’s own groups remained unchanged, thus making it possible to disentangle effects of income and inequality.

### Participants and Design

Participants were 151 MTurk workers (62 females, 89 males, with an average age of *M =* 32.02 years, *SD* = 9.50). The design was a 3 (wealth) x 2 (growing or declining inequality). Wealth was manipulated at three levels: as lower than average wealth (income group 2), moderate wealth (income group 3), and above average wealth (income group 4). Participants were randomly assigned to conditions.

### Ethics Statement

This study also obtained ethical clearance from the Behavioural and Social Sciences Ethical Review Committee (BSSERC) at the University of Queensland. Before completing the questionnaire, participants were informed about the aims of the study. After this, they were informed that continuing with the survey indicated (written) informed consent.

### Procedure and measures

As in Study 2, participants were told that they would become part of a hypothetical society, Bimboola. After they had been assigned randomly to one of three wealth groups in a five-income group society, they were again asked to buy items that would help them to get started in their new life. Participants were subsequently exposed to the growing or declining inequality manipulation. Specifically, in the declining inequality condition, participants were told (growing income inequality condition in brackets) “Imagine that over the next 20 years, Bimboola is affected by a change in economy. As a result, the wealth gap in Bimboolean society has decreased (increased). Status differences have decreased (increased): the poor have become richer (poorer), the moderately wealthy earn about the same, and the rich have lost some of the wealth and become poorer (gained more wealth and become richer).” After this, participants were also presented with a graphical representation of this change. Importantly, in the graph, the wealth of the own income group was presented as unaffected over time and this was emphasized in a clarifying note to the graph.

Participants then completed the same two-item wealth manipulation check as used in Study 2 (*r* = .69, *p* < .001). In addition, the growing versus declining inequality manipulation was checked with four items. On 7 point scales ranging from 1 = *Strongly disagree* to 7 = *Strongly agree*, participants were asked “Over time, the gap between the poor and the rich has become wider in Bimboola”, “Over time, the gap between the poor and the rich has become narrower in Bimboola”, “Over time, inequality between the income groups has increased in Bimboola”, and “Over time, inequality between the income groups has decreased in Bimboola”. After recording of the second and fourth item, responses on the four items were averaged with higher scores indicating growing inequality perceptions (α = .93). To ensure that our inequality manipulation had not also affected participants’ own wealth ratings, we added a question asking them to what extent they agreed that “the income of my group has stayed the same over time”.

After this, participants were again informed that a new group (called newcomers) would be joining Bimboola. After completing all measures, participants were debriefed, received a code they used for payment and they were thanked for their participation.

#### Opposition to immigration

To measure opposition to immigration by the newcomers, most of the items used in Study 2 were included and a few general opposition to immigration items were added, totalling 20 items. Responses were again measured on a 7 point scale from 1 = *Strongly disagree* to 7 = *Strongly agree* (α = .96).

### Results

#### Manipulation check

A 3 (wealth) x 2 (growing or declining inequality) ANOVA on the wealth check revealed a main effect for wealth, *F*(2, 147) = 72.81, *p*<. 001, η^2^ = .49. In line with the manipulations, above average wealth income group participants agreed most with the statement that their group was rich (*M* = 5.88, *SD* = 1.11), followed by moderately wealthy income group participants (*M* = 4.84, *SD* = .93), followed by poor income group participants (*M* = 3.18, *SD* = 1.47). Post-hoc tests confirmed that all conditions differed significantly from each other at *p* < .001. Albeit much weaker than the wealth condition effect, we also found a main effect for inequality, *F*(1, 148) = 4.04, *p* = .046, η^2^ = .03, whereby participants in the growing inequality condition felt wealthier (*M* = 4.82, *SD* = 1.56) than those is the declining inequality condition (*M* = 4.45, *SD* = 1.68). It is possible this effect is the result of the graph that participants were asked to study whereby there is a greater gap between the own group and the poorest income group (income group 1) in the growing than in the declining inequality condition. However, this unexpected effect is small, and may well be a fluke effect. The interaction between wealth and inequality was not significant, *F*(2, 147) = 2.14, *p* = .122, η^2^ = .03.

A similar ANOVA on the inequality check revealed only a main effect for inequality, *F*(1, 148) = 82.45, *p*< .001, η^2^ = .35. In line with the manipulation, participants perceived income inequality to be higher in the growing inequality condition (*M* = 5.24, *SD* = 1.38), than in the declining inequality condition (*M* = 3.06, *SD* = 1.64).

Finally, we found no significant effects of wealth or equality on the judgement whether wealth had stayed the same over time, all *F*s < 2.16, suggesting that the equality information had not affected perceptions of own wealth differently across the conditions. In line with the manipulations, participants perceived that their wealth had stayed the same (*M* = 6.09, *SD* = .97) despite changes in the equality of society. In general, we found that the manipulation of wealth and inequality worked as intended.

#### Opposition to immigration

**Fig 2:**
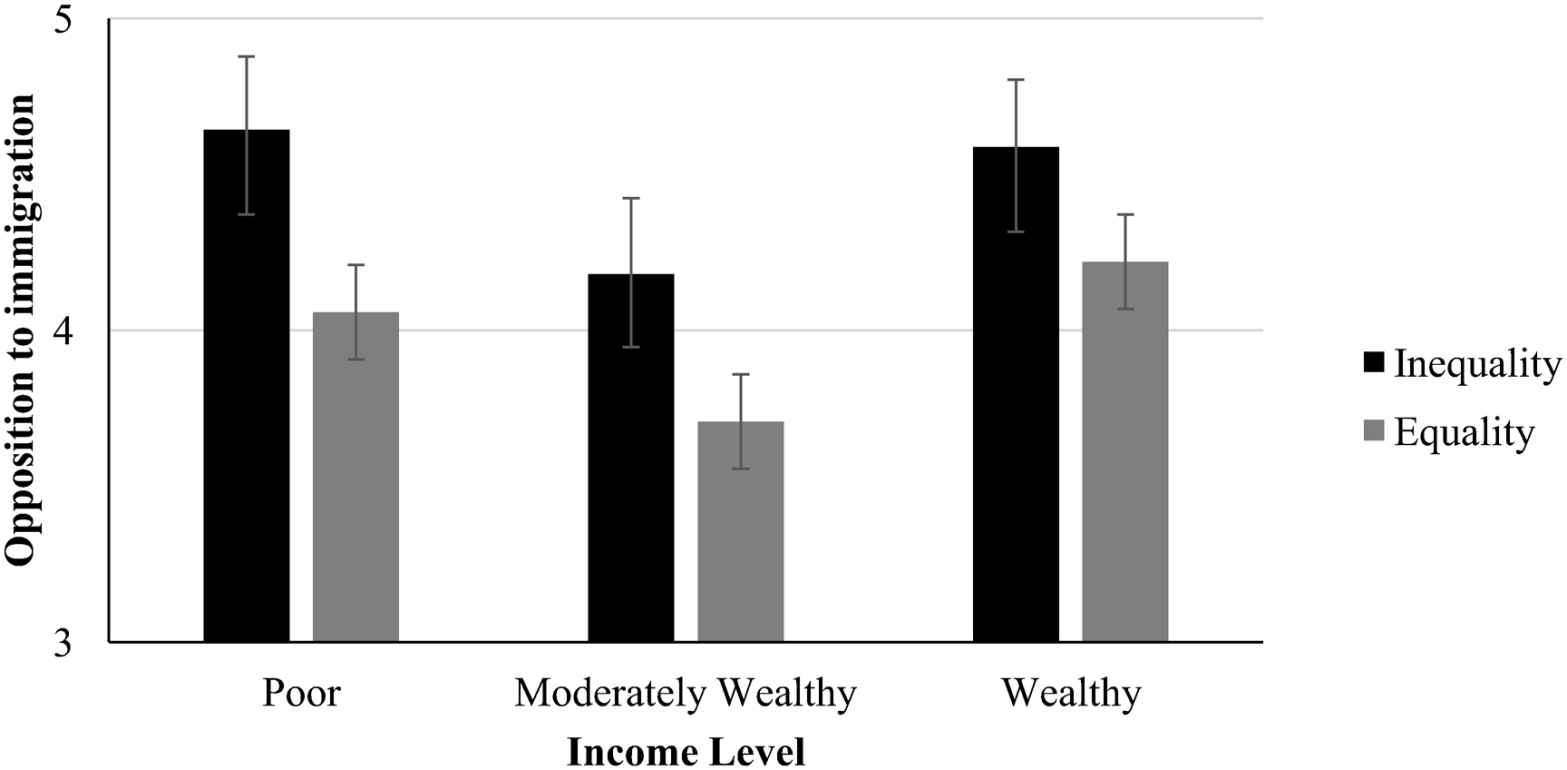
Opposition to immigration as a function of income level and societal inequality, Means and Standard Errors, Study 3.

Analysis of variance revealed that the linear effect for wealth was not significant, *F*(1,148) = .12, *p* = .78, η^2^ = .03, but the quadratic term was significant, *F*(1,148) = 5.34, *p* = .022, η^2^ = .04, providing evidence for a v-curve: Opposition to immigration was highest in the poor income group condition (*M* = 4.39, *SD* = 1.25), *and* the above average wealth group condition (*M* = 4.45, *SD* = 1.26). Opposition to immigration was lowest in the moderate wealth income group (*M* = 3.93, *SD* = 1.17). We also found a significant effect of inequality, *F*(1, 145) = 5.61, *p* = .019, η^2^ = .04. Opposition to immigration was higher in the growing inequality condition (*M* = 4.47, *SD* = 1.21) than in the declining inequality condition (*M* = 4.00, *SD* = 1.22). The interaction between income level and inequality was not significant, *F*(2, 145) = .10, *p* = .91 (see Fig 2).

### Discussion

In sum, Study 3 replicated Study 2 findings and showed that participants in the poor *and* above average wealth income groups were more opposed to immigration compared to those in a moderately wealthy group. We also found that societal equality affected opposition to immigration, but not in the way that we had expected. Whereas we had proposed that inequality would amplify the v-curve because in particular participants in poor and above average wealth conditions would perceive inequality as more threatening and anxiety provoking than those in the moderately wealth condition, we found instead that participants in *all* three wealth groups showed more opposition to immigration when their society was expected to become more unequal compared to equal. Even though this finding was not predicted, it is in line with observations by Wilkinson and Pickett [74] that inequality affects everyone in society—the poor as well as the wealthy. We will discuss this finding in greater detail in our General Discussion.

Finally, we also found that growing inequality led to a perception of being wealthier and overall greater opposition to immigration than in the declining inequality condition. Even though the higher wealth perceptions in unequal societies may be a result of the way inequality was manipulated, the latter finding on opposition to immigration is consistent with a growing body of work revealing the negative effects of societal inequality on tolerance and well-being more generally (e.g., [76, 74]).

## Study 4

Even though Studies 1 to 3 provide good evidence for a v-curve using both correlational and experimental designs, it is not clear yet (a) what forms of relative gratification are most conducive to promote opposition to immigration and (b) why this relationship emerges. These questions were the focus of the fourth and final study where we examined the relationship between perceptions of relative gratification and opposition to immigration in an Australian community sample. To address the first question, we examined a range of different indicators of relative gratification including personal level (i.e., gratification when considering own personal wealth), and collective level relative gratification (i.e., gratification when considering Australia’s wealth). In an attempt to assess whether effects of current relative gratification are similar to effects of past and expected future relative gratification, for both personal and collective level relative gratification, we also examined relative gratification relating to current wealth, current wealth compared to the past, and expected future relative gratification. At the personal level, we also included a measure of class as a relatively fixed indicator or relative gratification. Finally, at both the collective and personal level, we examine the extent to which participants felt relatively gratified when considering the threat posed by the Global Financial Crisis (GFC). More specifically, we focused on whether participants *minimized* the impact of the GFC both personally and at the country level, either because they or their country had recovered quickly from the GFC or had not been affected as much by it compared to other individuals or countries.

Another important aim of Study 4 was to examine processes underlying relative gratification. We examined the extent to which (1) identification, (2) collective self-definition as competent but cold, and (3) fear for future wealth can account for the relationship between past, present and future collective relative gratification and opposition to immigration.

### Method

#### Participants

Participants were 621 Australian residents (326 females, 295 males) with an average age of 40.90 (*SD* = 12.78, 7 missing values). In terms of ethnicity, 76.9% reported to be White Caucasian, 15.8% were Asian, 1.4% were Middle Eastern, .8% were Aboriginal and/or Torres Strait Islander, and 3.9% ticked the ‘other’ category. To the question “what is your highest level of education”, 89 indicated they did not complete year 12, 101 completed year 12, 146 had a post-secondary qualification, 155 had finished an undergraduate degree and 117 participants held postgraduate qualifications (13 preferred not to say). Analyses revealed that controlling for ethnicity or education did not change the interpretation of the results reported below.

#### Ethics Statement

This study also obtained ethical clearance from the Behavioural and Social Sciences Ethical Review Committee (BSSERC) at the University of Queensland. Before completing the questionnaire, participants were informed about the aims of the study. After this, they were informed that continuing with the survey indicated (written) informed consent.

#### Procedure and Measures

The study was part of a larger survey examining participants’ perceptions of their life in Australia and their views of Australian society more generally. Here, we focus on a subset of measures. A professional research company (Taverner Research based in Sydney) collected the data. Participants were sampled from the company’s research panel, which covered all Australian states and territories.

#### Collective relative gratification

Collective relative gratification was measured with a number of items that tapped different forms of relative gratification relating to collective wealth. Specifically, we measured current, past and future gratification at the collective level using the items: “Please think of the economic situation in Australia at the moment. How would you describe the current economic situation in Australia?”, “Now please think about Australia’s economic situation 3 years ago. To what extent would you describe Australia’s economic situation three years ago to be worse, the same, or better than it is now? “and “Now think about Australia’s economic situation in the next 3 years. To what extent do you expect Australia’s economic situation to be worse, the same, or better in the next 3 years?” Responses to the first item were recorded on a scale ranging from 1 = *Very bad*, to 7 = *Very good*. The scale for the second and third item assessing *past collective gratification* and *future collective gratification* ranged from 1 = *A lot worse*, to 7 = *A lot better*. Two items were included asking about the extent Australia was unaffected and had recovered well from the Global Financial Crisis: “Our country has already recovered quite well from the negative impact of the global financial crisis”and “The effect of the global financial crisis on our country’s economy has been negligible” (*minimize impact of GFC*, 1 = *Strongly disagree* to 7 = *Strongly agree*, *r* = .49, *p* < .001).

#### Personal relative gratification

Personal relative gratification was measured by asking participants to indicate their level of satisfaction with their current personal financial situation with the item “Please think of your personal economic situation at the moment. How would you describe your current economic personal economic situation?” (*current personal gratification*, 1 = *Very bad*, to 7 = *Very good*). We also asked participants about past and future personal gratification with the items “Now please think at your personal economic situation 3 years ago. To what extent would you describe your personal economic situation 3 years ago to be worse, the same, or better than it is now?” and “Now think about your personal economic situation in the next 3 years. To what extent do you expect your personal economic situation to worsen, remain the same, or improve in the next 3 years?” (*past personal gratification* and *future personal gratification*, 1 = *A lot worse*, to 7 = *A lot better*). Finally, two questions asking about relative gratification as being personally unaffected or having recovered well from the Global Financial Crisis: “The effect of the global financial crisis on my personal economic situation has been negligible “and “I have personally already recovered quite well from the negative impact of the global financial crisis” (*minimize impact of GFC*, 1 = *Strongly disagree* to 7 = *Strongly agree*, *r* = .47, *p* < .001). We also added one item asking about participants’ social class: “How do you perceive your own family’s class/social background?” (1 = *Lower class* to 7 = *Upper class*). As a measure of personal wealth, we also asked participants to record their annual household income. However, 40.5% of respondents indicated that they did not know or did not want to provide this information. Given the high number of missing values, we decided not to analyze responses to this measure.

#### Group identification

Four items, adapted from Doosje, Ellemers, and Spears [77] were used to measure identification with Australia (7 point scale from 1 = *Strongly disagree* to 7 = *Strongly agree*). Higher values indicated more identification with the own income group (α = .95).

#### Collective self-definition as competent and cold

Participants were asked to indicate on a 7-point scale from 1 = *Strongly disagree* to 7 = *Strongly agree* the extent to which they perceived Australians to be warm (“warm”, “friendly”, “likeable”, “harsh”, “ruthless”, reverse coding of the last two items), and competent [“competent”, “capable”, “weak” (reverse-coded)]. The reliability of the warmth (α = .74), and competence scales (α = .64) was satisfactory.

#### Fear about future wealth

One item, adapted from Jetten and Wohl [78] was included to gauge fear about future wealth at the collective level: “I feel anxious about the future wealth of Australia”. We also included the other items of the collective angst scale that this item is taken from. Similar effects were found for this scale as for the one item measure assessing fear about the future wealth of the country.

#### Opposition to immigration

Opposition to immigration was measured using 6 items assessing both realistic threat perceptions (e.g., “Immigrants take resources and employment opportunities away from Australians”) and symbolic threat perceptions (e.g., “The cultural practises of immigrants threaten the Australian way of life”). Responses were recorded on 7 point scales ranging from 1 = *Strongly disagree* to 7 = *Strongly agree*. Participants did not differentiate between realistic and symbolic threat and we combined the items to form one scale, α = .95.

### Results

A series of curve estimation analyses were conducted exploring the extent to which each of the collective and personal level relative gratification measures predicted opposition to immigration (see Table 2). Given the fact that we conducted multiple tests, a conservative significance level of *p* < .01 was used.

**Table 2:** Results of curve estimation analyses predicting opposition to Immigration, and examinations of potential mediators, Study 4.

Predicting opposition to immigration		Linear β	Quadratic β		
Collective level Relative Gratification as predictor				Linear β	Quadratic β
	*M (SD)*				
Collective Relative Gratification					
Current	3.92 (1.37)	-.17**	.47*		
Past	4.29 (1.32)	-.04	.46		
Future	3.80 (1.39)	-.08	.73**		
Minimize impact of GFC	4.31 (1.20)	.07	.49*		
Personal Relative Gratification					
Current	3.95 (1.53)	-.10*	.65**		
Past	4.39 (1.39)	-.04	.43		
Future	4.18 (1.39)	-.18**	.51**		
Minimize impact of GFC	4.11 (1.21)	.00	.38		
Class	4.09 (1.22)	-.05	.26		
Identification	5.54 (1.35)	.11*	-.10	.16**	-.10
Collective self-definition as warm	4.78 (.92)	.09	-.36	.00	-.36
Collective self-definition as competent	5.00 (1.00)	.13**	.01	.04	.01
Fear for future wealth	4.86 (1.41)	-.23**	.76**	.26**	.76**

*p< .01

**p < .001

For the quadratic analyses, a positive beta indicates a v-curve, a negative beta indicates an inverse v-curve.

Inspection of the linear and quadratic effects showed a number of significant relationships. First, focusing on the collective relative gratification predictors of opposition to immigration, only higher current collective relative gratification was linearly associated with lower opposition to immigration, *β* = -.17, *p* < .001. There was evidence for curvilinear relationships, such that opposition to immigration was higher among those with lower and among those with higher wealth levels (current collective gratification, *β* = .47, *p* = .01, future collective gratification, *β* = .73, *p* < .001). Interestingly too, the quadratic term of minimizing the impact of the GFC on Australia also predicted opposition to immigration: it was those who felt Australia had not recovered from the GFC (relative deprivation) and those who felt Australia was hardly affected by the GFC (relative gratification), minimizing the effects of the GFC that was associated with higher opposition to immigration, *β* = .49, *p* = .007.

There were also a number of significant relationships between different indicators of personal relative gratification and opposition to immigration. Specifically, higher current and future personal relative gratification was linearly associated with lower opposition to immigration, *β* = -.10, *p* = .01 and *β* = -.18, *p* < .001, respectively. In both cases, the quadratic effect was also significant. Similar to the collective level analyses, and in line with the v-curve hypothesis, higher opposition to immigration was found for those that indicated lower and higher current and future wealth, *β* = .65, *p* < .001 and *β* = .51, *p* = .002, respectively. Minimizing the personal effects of the GFC or class did not predict opposition to immigration, neither linearly nor curvilinearly (see Table 2).

#### Mediators

A further series of curve estimation analyses were conducted examining the power of (1) identification, (2) collective self-definition as competent and cold, and (3) fear for the future wealth of Australia to explain the relationship between collective relative gratification and opposition to immigration. These analyses were conducted using only present collective relative gratification measures. Results of these analyses are reported in Table 2.

Inspection of the linear relationship between collective relative gratification and each of the mediators revealed a significant linear relationship for identification, *β* = .11, *p* = .006, collective self-definition as competent, *β* = .13, *p* = .001, and fear for future wealth, *β* = -.23, *p* = .001. Collective relative gratification was associated with higher identification, a higher collective self-definition as competent and less fear for future wealth. Inspection of the curvilinear relationships revealed only a significant effect for fear for future wealth, *β* = .76, *p* < .001. In other words, it was those who were highest and those who were lowest in collective gratification that feared most for the future wealth of Australia.

Further analyses revealed that only a few potential mediators predicted opposition to immigration. Higher identification was linearly related to greater opposition to immigration, *β* = .16, *p* < .001, and higher fear for the future wealth of Australia was linearly associated with higher opposition to immigration, *β* = .26, *p* < .001.

Because only national identification (linearly) and fear for future wealth (linear and quadratic) met the conditions for mediations, it was only these variables that were included as mediators in bootstrap analyses [79] using 5000 resamples. This revealed that group identification was not a significant mediator (IE = .03, *SE* = .012, 95% CI[-.003, .004]). However, fear for future wealth (linear) turned out to be a significant mediator (IE = -.07, *SE* = .018, 95% CI[-.121, -.107]). Importantly too for our purposes, the quadratic future wealth term was also a significant mediator (IE = -.07, *SE* = .019, 95% CI[-.126, -.111]), indicating that collective gratification predicted opposition to immigration because, relatively speaking, it was those who were highest and lowest in collective gratification that feared most for the future wealth of their country.

### Discussion

In sum, even though we found an overall negative relationship between relative collective gratification and opposition to immigration in Australia, in addition to these linear effects we found the predicted v-curve relationship between relative gratification and opposition to immigration. More specifically, we found evidence of a v-curve whereby the relationship between perceived relative gratification and opposition to immigration was most pronounced for those who felt collectively relatively deprived *or* relatively gratified at present or expected to feel so in the future. A similar v-curve was found for the relationship between personal relative gratification and opposition to immigration: it was those who felt relatively poor or wealthy at present or in the future who were more opposed to immigration than those who felt moderately wealthy. Interestingly too, we found that relative gratification by minimizing the negative effects of the GFC on Australia also produced the predicted v-curve relationship. Oppose to immigration to Australia was relatively high among those who denied and those who endorsed the statement that these negative effects were no longer of concern.

Study 4 also provided evidence for the underlying processes. At the collective level, it was only fear for the future wealth of Australia that mediated the relationship between collective gratification and opposition to immigration. Interestingly, the relationship between collective gratification and fear for the future wealth of Australia was curvilinear: it was those lower and higher in relative gratification who feared future wealth decline of Australia. This finding is consistent with findings by Moscatelli and colleagues [66] and suggests that relative gratification, because it is associated with relative fear for the future wealth of Australia, predicts opposition to immigration.

## General Discussion

We set ourselves the task of exploring whether there is evidence for a v-curve shaped relationship between the economic performance of a group or society and opposition to immigration. That is, we predicted that opposition to immigration would be highest among groups that are relatively deprived and poor as well as among those with above average wealth (i.e., relatively gratified). We found evidence for such a relationship in four studies that examined support for the v-curve in different contexts and using different research designs.

In Study 1, we found that voting in favour of a national policy to curb immigration was highest in Swiss cantons where unemployment was lowest and highest (a v-shaped curvilinear relationship). Even though we only found a positive linear relationship and not the predicted curvilinear relationship between canton relative disposable income and voting in favour of this anti-immigration policy, the failure to find evidence for a v-curve was due to the failure to find greater support for this policy among the poorest cantons, not because of the lack of support for higher levels of endorsement of the policy in the wealthier cantons. Even though this finding supports relative gratification theorizing [9, 11] it is at odds with classic relative deprivation reasoning [15, 17].

Studies 2 and 3 examined the v-curve using an experimental design. Here too, we found that in both studies opposition to ‘newcomers’ joining a hypothetical society was higher among poor and above average wealth group members than among those in a moderate wealth group condition. Finally, in a cross-sectional study, in line with the v-curve hypothesis, opposition to immigration spiked for those who experienced lower and higher personal or collective levels of current and future wealth. We also found a v-curve between minimizing the effects of the GFC at the collective level and opposition to immigration. In sum, across the four studies, we found consistent support for the v-curve, whereby both wealth deprivation and wealth gratification are associated with greater support for policies aimed at curbing immigration (Study 1), greater opposition to immigration by newcomers to a hypothetical society (Studies 2 and 3), and greater opposition to immigrants seeking to migrate to Australia (Study 4).

### Implications

In order to better understand the processes underlying the v-curve and the conditions under which outgroup hostility will be most pronounced, it is useful to review other findings that emerged from the studies. First, in our examination of the way societal inequality affects the v-curve (Study 3), we did not find (as predicted) that the v-curve would be more pronounced with growing than with declining inequality. Instead, in addition to a v-curve, we found that *all* wealth groups became more opposed to immigrants when inequality was growing rather than declining. This finding is interesting and suggests that growing inequality is equally threatening for those at the bottom, middle or top of a wealth hierarchy. The finding is consistent with observations by political scientists and sociologists that growing inequality leads to greater status competition whereby *everyone* experiences greater status instability and status anxiety [50,74].

Whereas the finding for growing inequality effects is interesting and consistent with Postmes and Smith’s findings ([11], Study 2), equally interesting (and inconsistent with ([11], Study 2) is that we still found evidence for a v-curve among participants who were informed that inequality in their society was declining. It may be that the way we manipulated declining inequality is responsible for this finding. In our declining inequality condition, income differences between groups became smaller and this may have threatened distinctiveness between the income groups. There is a large body of work showing that threats to distinctiveness leads to a motivation to restore intergroup distinctiveness, often by showing enhanced intergroup discrimination towards other groups [80–82]. The extent to which distinctiveness threat associated with smaller differences between income groups enhanced perceived competition for those who felt relatively gratified should be examined in future research.

Second, in an attempt to better understand what forms of relative gratification are associated with opposition to immigration, in Study 4 we examined the relationship between collective and personal relative gratification as well as the relationship between current, expected future and past relative gratification with opposition to immigration. Interestingly, past relative gratification (i.e., perceiving that personal and collective wealth was higher in the past than in the present) did not predict opposition to immigration. However, and in line with the v-curve hypothesis, opposition to immigration was highest for those who experienced high personal and/or collective levels of current and future relative gratification. We can only speculate why current and expected relative gratification predicted opposition to immigration whereas temporal comparisons with past wealth did not. We suspect that a sense of current and expected future gratification is associated with greater wealth stability [70] and that this may feed into a sense of deservingness or entitlement [58, 59] thereby justifying the exclusion of immigrants. In contrast, awareness that one was less wealthy in the past than the present may evoke a sense of humbleness, reminding people that they were once less well-off themselves, and this should attenuate hostility and prejudice. However, these are speculative suggestions that should be tested in future research.

We also found that relative gratification emerging from a sense that one’s country is relatively more robust against the global financial crisis than other countries also predicted opposition to immigration. This should serve as a reminder that it is not so much absolute wealth, but feeling wealthier than others that matters when predicting outcomes of relative gratification [65, 9].

It is interesting that we found that both personal and collective level gratification predicted opposition to immigration. Even though this appears to go against classic reasoning that collective level outcomes are predicted by collective level processes relating to fraternalistic instead of egoistic deprivation or gratification perceptions [71, 8], it may well be that these judgements are not always all that different. Indeed, because self-definition is in important ways defined by group membership, perceptions of personal achievement are often bound up with the position of ones group in society [70, 8].

Finally, Study 4 provided insight in the processes underlying the relationship between relative gratification and opposition to immigration. Even though self-definitions as competent predicted more opposition to immigration, we did not find that relative gratification was associated with greater competence. We also found no support for the idea that self-definitions as competent triggered self-definitions of being cold. Indeed, self-definitions as warm/cold were not related to either relative gratification or opposition to immigration. It may well be the case that we would find more support for a mediating role of self-definitions as competent and cold in context where wealth is attributed to competence (e.g., within organisations or when people are able to secure upward mobility by joining higher classes in society). We suspect that under such conditions wealth may trigger feelings of deservingness and entitlement and these may also be the conditions where groups will self-define as cold, both providing some moral justification for holding negative attitudes of immigrants. This should be examined in future research.

We found no support for the prediction that group identification mediates the relationship between relative gratification and opposition to immigration (contrary to [9]). Even though relative gratification predicted identification with Australia linearly, and identification, in turn, predicted higher opposition to immigration, bootstrapping analysis showed that identification did not mediate this relationship. Theoretically, this is not surprising. The prediction that greater commitment to the group enhances opposition to immigration is based on the assumption that group identification is positively correlated with ingroup bias and outgroup prejudice. While this may be true in certain contexts, some have warned against assuming a straightforward relationship between the two [83]. For example, there is evidence that group identification only promotes ingroup bias to the extent that this is normatively prescribed in a group [81].

We did however find that fear for future wealth mediated the relationship between relative gratification and opposition to immigration and accounted for the observed v-curve. What this suggests is that relative gratification is associated with fear that future wealth may be lost and it is this ‘fear of falling’ notion [69] that helps to explain why those who feel relatively gratified become more negative towards minorities such as immigrants. Opposition to immigration thus results from anxiety and fear that the relative gratified position may be lost in the future, and this undermines the security of the wealth position. Our findings thus echo research showing that for high status groups, status instability and insecurity can lead to a stress response [56] and increased discriminatory behaviour in intergroup settings, ([46], see also [55]).

### Limitations and future research

Our studies are not without limitations and they raise new questions to be examined in future research. First, we suggest that it may be instructive to examine the effects of societal inequality in greater depth in future research. Interestingly, our findings suggest that everyone becomes harsher when income inequality grows compared to when income inequality declines. However, because we needed to ensure that the income of the own wealth group did not change with growing or declining inequality (so that our wealth manipulation would not be confounded with our inequality manipulation), our inequality manipulation may also have been rather weak. This is because the stability of the own group wealth may have taken away some of the uncertainty that everyone experiences when boundaries between groups become more permeable due to growing inequality, and when all wealth groups start to fear for their future wealth. This too should be explored in future work, whereby societal inequality should be manipulated in different ways.

It is also essential in future research to examine the processes underlying growing societal inequality effects. Indeed, while the effects of inequality have been well documented [74], less progress has been made in understanding *why* inequality is associated with such negative outcomes. The paradigm that was presented in Studies 2 and 3 might be useful to examine these underlying processes experimentally.

As a final suggestion for future research, we note that we found evidence for the v-curve in contexts dominated by relative gratification relationships in Study 1 (i.e., there was a linear positive relationship between relative disposable income and anti-immigrant votes) but relative deprivation relationships in Study 4 (i.e., a negative linear relationship between current and future collective relative gratification and opposition to immigration). Put differently, evidence for the v-curve was found against the backdrop of a general relative gratification effect (Study 1) *and* a general relative deprivation effect (Study 4). This suggests that in most contexts, multiple processes may be at play at once, simultaneously co-determining the relative strength of prejudice and hostility due to relative deprivation (e.g., realistic conflict, competition over resources) as well as relative gratification related processes (e.g., anxieties and fear that wealth will be lost). These processes may differ from laboratory to natural context due to a host of extraneous factors, making it very difficult to predict a priori which process will dominate or to make strong claims about the generalizability of findings. This reasoning is consistent with observations that there is no straightforward relationship between economic downturns and opposition to immigration [43, 31, 13] and that what is required is great sensitivity to the context and the socio-structural context that may help to inform researchers which processes will dominate. But notwithstanding these variations in the tilt of the v-curve pattern across contexts, it should be noted that in none of these studies was the curvilinear effect reversed. In other words, we are satisfied with the robustness of the underlying effect.

### Final thoughts

Although our work is not the first to demonstrate the v-curve empirically, by examining this relationship in new ways, we have advanced our understanding of the way economic performance of a group affects opposition to immigration. We contribute to this body of work by providing correlational as well as experimental evidence for the v-curve, by examining different forms of relative gratification and by providing greater clarity on the processes underlying the relationship between relative gratification and opposition to immigration. Although wealth undoubtedly reduces certain kinds of stress, we find that relative gratification and wealth also triggers fears that wealth will be lost in the future. Ironically, then, the perception that one is relatively wealthy is therefore not always more comfortable than the perception that one is relatively deprived. In some sense, relative gratification and relative deprivation are not the polar opposites they might seem to be. Feelings of gratification might be pleasing and comfortable at one level, but like all delights such relative advantages might well bring latent insecurities of loss and fears of decline.
